# Trends in Consumption of Solid Fats, Added Sugars, Sodium, Sugar-Sweetened Beverages, and Fruit from Fast Food Restaurants and by Fast Food Restaurant Type among US Children, 2003–2010

**DOI:** 10.3390/nu8120804

**Published:** 2016-12-13

**Authors:** Colin D. Rehm, Adam Drewnowski

**Affiliations:** Center for Public Health Nutrition, University of Washington, Box 353410, Seattle, WA 98195, USA; crehm@uw.edu

**Keywords:** energy intake, dietary surveillance, food analysis/methods, nutritive value, dietary sucrose, dietary fats, fruit

## Abstract

Energy intakes from fast food restaurants (FFRs) have declined among US children. Less is known about the corresponding trends for FFR-sourced solid fats, added sugars, and sodium, and food groups of interest, such as fruit and sugar-sweetened beverages (SSBs). Using data from a single 24-h dietary recall among 12,378 children aged 4–19 years from four consecutive cycles of the nationally-representative National Health and Nutrition Examination Survey (NHANES), 2003–2010 a custom algorithm segmented FFRs into burger, pizza, sandwich, Mexican cuisine, chicken, Asian cuisine, fish restaurants, and coffee shops. There was a significant population-wide decline in FFR-sourced solid fats (−32 kcal/day, *p*-trend < 0.001), added sugars (−16 kcal/day; *p*-trend < 0.001), SSBs (−0.12 servings (12 fluid ounces or 355 mL)/day; *p*-trend < 0.001), and sodium (−166 mg/day; *p*-trend < 0.001). Declines were observed when restricted to fast food consumers alone. Sharp declines were observed for pizza restaurants; added sugars, solid fats, and SSBs declined significantly from burger restaurants. Fruit did not change for fast food restaurants overall. Temporal analyses of fast food consumption trends by restaurant type allow for more precise monitoring of the quality of children’s diets than can be obtained from analyses of menu offerings. Such analyses can inform public health interventions and policy measures.

## 1. Introduction

Energy intakes from fast food restaurants (FFRs) among US children have declined from 2003 and 2010 [1]. Less is known about the corresponding consumption trends for key nutrients and food groups, including solid fats, added sugars, sodium, sugar-sweetened beverages, and fruit [2]. While FFRs contributed an average of 14.1% of energy to the children’s diet (aged 4–19 years), they accounted for 17.9% of solid fats, 10.4% of added sugars, and 15.9% of dietary sodium [3]. Additional insights into children’s diets can be provided by examining nutrient consumption patterns by the type of fast food restaurant [3].

Since 2003–2004, all foods and beverages in the National Health and Nutrition Examination Survey (NHANES) have been identified by their location of origin. Among identified food purchase locations were fast food (FFR) and full service restaurants (FSR). The type of FFR was not specified. We have developed a custom algorithm to assign all FFR-identified meals and snacks into distinct categories by FFR type [3]. Following industry segmentation practice, FFR categories were defined as burger, pizza, sandwich, chicken, Mexican, Asian, and fish restaurants, and coffee shops [3]. 

Based on published analyses of NHANES 2003–2010 data [3], burger restaurants were the leading market segment, providing 6.2% of total energy in the children’s diet. They were followed by pizza (3.3%), sandwich (1.4%), Mexican (1.3%), and chicken restaurants (1.2%). Examining data per capita and per consumer showed that the observed decline in mean energy intakes could be due to fewer children consuming fast food on any given day and/or to fewer calories consumed per eating occasion [1].

The present descriptive study examined consumption trends in FFR-sourced solid fats, added sugars, and sodium from 2003–2010. Dietary Guidelines for Americans (DGAs) have identified solid fats and added sugars as the chief sources of “empty calories” in the US diet [4]. Also of interest was sodium consumption, given concerns that about 90% of US children consume excess sodium [5]. Time trends in sugar sweetened beverages (SSBs) consumption were also examined and contrasted to trends in fruit consumption, including whole fruit and 100% fruit juice [6,7,8]. Analyses were conducted for the total population of children (per capita) and for those children who were FFR consumers (per consumer).

## 2. Methods

### 2.1. Dietary Intake Data

Data analyses were based on the first 24-h recall from four cycles (2003–2004, 2005–2006, 2007–2008, and 2009–2010) of the nationally representative National Health and Nutrition Examination Survey (NHANES). The necessary ethical approvals for NHANES have been obtained by the National Center for Health Statistics and the data are publicly available via the NCHS website [9,10]. In the NHANES 24-h multi-pass method, all foods and beverages consumed in the preceding 24-h were reported, from midnight to midnight. The time of each eating occasion were also obtained. For children 4–5 years, the dietary recall was completed by a parent/guardian. For children 6–11 years, the child was the primary respondent, but the parent was present and able to assist. For children 12–19 years, the child was the primary respondent, but could be assisted by an adult [11].

Data from the MyPyramid Equivalents Database (MPED) were used to assess intakes of added sugars and solid fats [12]. The MPED 2.0 database was updated for use with more recent NHANES cycles by imputing the MPED equivalents for a limited number of foods (*n* = 291). Energy from solid fats was estimated as 9 kcal per gram of solid fat. Solid fats refer to fats that are solid at room temperature, and include butter, fat from animal sources other than fish, stick margarines, shortening, hydrogenated vegetable oils, coconut, and palm oils [12]. Fats that are liquid at room temperature are considered oils in MPED, and are not included in the solid fats definition. In general, the MPED database assumed that French fries were cooked in beef tallow or using hydrogenated fats [12]. Since the MPED database provides added sugars in teaspoon equivalents, this value was converted to energy (1 tsp = 16 kcal) [13]. The database was developed prior to the release of the Food Patterns Equivalent Database (FPED), but values for added sugars and solid fats for the two databases for key foods consumed at fast food restaurants were highly comparable. Additional data were obtained for sugar-sweetened beverages (SSB), defined as beverages within the following categories containing more than 50 kcal per 236.6 mL or 8 fluid ounces: carbonated soft drinks/soda, fruit drinks, pre-sweetened iced tea, sports drinks, and energy drinks. Flavored milks were not included as SSBs. Fruit consumption was estimated based on values from MPED 2.0 and the fruit juice addendum provided by the United States Department of Agriculture Center for Nutrition Policy and Promotion, as the MPED database did not disaggregate between whole fruit and fruit juice. While most fruit juice in the MPED is 100% fruit juice, a small proportion comes from other recipes or combinations of fruit drinks with fruit juice.

The five locations of origin for all foods and beverages were obtained from NHANES data. These were: stores (including grocery stores and convenience stores), fast food restaurants (FFR), full-service restaurants (FSR), school cafeterias, and other [11]. Since NHANES data does not segment the FFRs category further, custom algorithms were developed to distinguish among different types of FFRs.

### 2.2. Types of Fast Food Restaurants

A multi-step algorithm was developed to assign FFR meals and snacks into one of eight types of fast food restaurants, as identified by the industry and published online [14]. The eight FFR segments were burgers (e.g., McDonald’s or Burger King), pizza (e.g., Pizza Hut or Domino’s), sandwich (e.g., Subway or Quiznos), chicken (e.g., KFC or Chick-fil-A), Mexican (e.g., Taco Bell), Asian (e.g., Panda Express), coffee/snack (e.g., Starbucks or Baskin-Robbins), or fish (e.g., Long John Silver’s). NHANES data include FFR meals from national, regional, and local establishments. 

FFR meals were assigned to one of the eight pre-defined segments based on the foods and beverages consumed at that meal. First, unique meals were defined as eating occasions that occurred at the same time and at the same location. Second, an iterative algorithm scanned the 24-h recall individual foods file for “sentinel” foods that could help identify a specific market segment (e.g., burger was a sentinel food for the FFR segment). Among the 26 sentinel foods were burgers, pizza, Mexican dishes, chicken strips/nuggets, fried chicken, submarine/deli sandwiches, and hot breakfast items as well as pretzels, hot dogs, fried chicken sides with no fried chicken (e.g., biscuits, potato salad), French fries alone, or soda alone.

Foods and food combinations that were representative of a FFR category (e.g., hamburger, fried chicken, or pizza) were identified. If a given meal occasion included only one of these sentinel main dishes, it was coded as such. Meals that contained multiple potential main dishes were flagged for further scrutiny. The initial scan coded 77.3% of single items, while the remainder were assessed manually. A small proportion of eating occasions could not be unambiguously assigned to a specific FFR category, and was coded based on the relative market share. 

For some meals, the assignment to a FFR segment was more complex. For example, chicken nuggets/strips are typically sold at both burger and chicken FFRs. Other examples include soda consumed alone, hot breakfast dishes, French fries alone, or ice cream. These meals were randomly assigned to each segment according to a deterministic probability based on weights from sales data for the 50 largest chains.

The reliability of the algorithm was evaluated by an independent coder who assigned 138 random meals (412 individual food items) into the eight FFR segments. The Kappa (chance-corrected concordance) was 0.89 comparing the algorithm to the hand coding, indicating a high level of agreement. Some refinements were made to the algorithm following the reliability study, including the removal of sweet and sour sauce as a sentinel food for the Asian category, since most sweet and sour sauce in NHANES was consumed with chicken nuggets/strips.

### 2.3. Analytical Approach

NHANES 2003–2010 data for 12,378 children and adolescents were analyzed [7]. First, trend analyses by two-year cycles examined the consumption of total and “empty” calories and sodium by purchase location. Two summary measures were assessed: the survey-weighted mean and the survey-weighted population proportion. The population proportion is the percent of each dietary constituent provided by each location of origin [15]. A linear trend test was calculated by treating the survey cycle as a continuous variable with the dietary variable of interest as the outcome in survey-weighted linear regression models. For analyses by fast food segment, separate analyses were conducted for the entire population (the per capita approach) and for consumers of each type of FFR (the per consumer approach). All analyses accounted for the complex survey design of NHANES data. Data analyses were conducted using Stata 13.1 (StataCorp 2015, College Station, TX, USA).

## 3. Results

### Time Trends 2003–2010 by Food Location of Origin

Table 1 shows trends for survey-weighted means and population proportions for energy, solid fats, added sugars, empty calories (solid fats + added sugars), sodium, sugar-sweetened beverages, whole fruit, and fruit juice by purchase among children aged 4–19 years. From all sources, total energy intakes declined by 205 kcal/day, driven by significant decreases from FFRs (109 kcal/day), grocery stores (86 kcal/day), and FSRs (40 kcal/day). Calories from FFRs declined from 341 kcal/day to 232 kcal/day, representing a shrinking proportion of the total: 15.6% to 11.7% of total (*p*-trend < 0.001). Although calories from grocery stores also declined (1423 kcal/day to 1337 kcal/day), they represented a higher proportion of the total in 2009–2010 compared to 2003–2004: from 64.8%–67.2% (*p*-trend = 0.011). Energy intakes from schools and other sources remained constant.

Energy from solid fats declined from 440 kcal/day to 391 kcal/day overall (*p*-trend < 0.001). There was a significant decline for FFRs (89 kcal/day to 57 kcal/day; *p*-trend < 0.001) and FSRs (30 kcal/day to 22 kcal/day; *p*-trend = 0.044), but not for grocery stores (261 kcal/day to 246 kcal/day; *p*-trend = 0.051).

Energy from added sugars declined from 389 kcal/day to 309 kcal/day (*p*-trend < 0.001). Significant decreases were observed for grocery stores (*p*-trend < 0.001), FFRs (*p*-trend < 0.001) and FSRs (*p*-trend = 0.031), but not for schools or other sources. 

The modest decline in sodium from 3313 mg/day to 3210 mg/day was not statistically significant (*p*-trend = 0.09). Significant decreases were observed for both FFRs (from 594 mg/day to 428 mg/day; *p*-trend < 0.001) and FSRs (from 261 mg/day to 164 mg/day; *p*-trend = 0.011). The contribution of FFRs to total sodium dropped from 17.9% to 13.3% of total (*p*-trend < 0.001). By contrast, there was no change in sodium sourced from grocery stores or schools and a significant increase in sodium from other sources (*p*-trend = 0.005).

In 2009–2010, store-bought foods were the dominant sources of energy in children’s diets, providing the bulk of calories (67.2%), solid fats (62.8%), added sugars (70.3%), and sodium (65.5%). FFRs contributed 11.7% of calories and FSR another 4.6%. On the average, FFRs supplied 6.3 g/day of solid fats (14.4%), 6.0 g/day of added sugars (8.2%), and 428 mg/day of sodium (13.3%).

Overall, sugar-sweetened beverages (SSBs) declined significantly from 1.63 serving (355 mL or 12 fl oz)/day to 1.16 serving/day (*p*-trend < 0.001. SSBs declined from all sources, except for other sources (e.g., not stores, restaurants or schools). The relative decline in SSBs was strongest for fast food restaurants (from 0.24 servings/day to 0.12 servings/day). Whole fruit increased significantly, while fruit juice declined significantly. For both, stores, followed by schools, were the predominant sources. Overall, fast food and full-service restaurants contributed little fruit to the diets of American children. 

Table 2 shows that FFRs contributed more calories to the diets of teenagers as compared to younger children. In 2009–2010, FFRs accounted for 9.4% of energy among 4–11 years old and 13.5% of energy for 12–19 years old, down from 13.7% and 17.1%, respectively, in 2003–2004. In 2009–2010, FFRs contributed 13.8% solid fats among 4–11 years old and 22.7% among 12–19 years old, which was statistically lower (*p* < 0.001) than 17.4% and 23.9%, respectively, in 2003–2004. Similar results were observed for added sugar and sodium. FFRs accounted for 8.0% of added sugar among 4–11 years old and 9.2% for 12–19 years old, down from 9.9% and 11.8%, respectively, in 2003–2004. For sodium, the drop was to 11.6% (2009–2010) from 15.6% (2003–2004) for 4–11 years old (*p*-trend = 0.018), and to 17.0% (2009–2010) from 20.9% (2003–2004) for 12–19 years old (*p*-trend = 0.006).

Analyses restricted to consumers of fast food were also conducted (Table 3). Significant declines were observed for energy (from 881 kcal/day to 714 kcal/day), solid fats (229 kcal/day to 174 kcal/day), added sugars (106 kcal/day to 77 kcal/day), empty calories (335 kcal/day to 251 kcal/day), sodium (1534 mg/day to 1315 mg/day) and sugar-sweetened beverages (0.62 servings (12 fluid ounces or 355 mL)/day to 0.36 servings (12 fluid ounces or 355 mL)/day).

**Figure 1:**
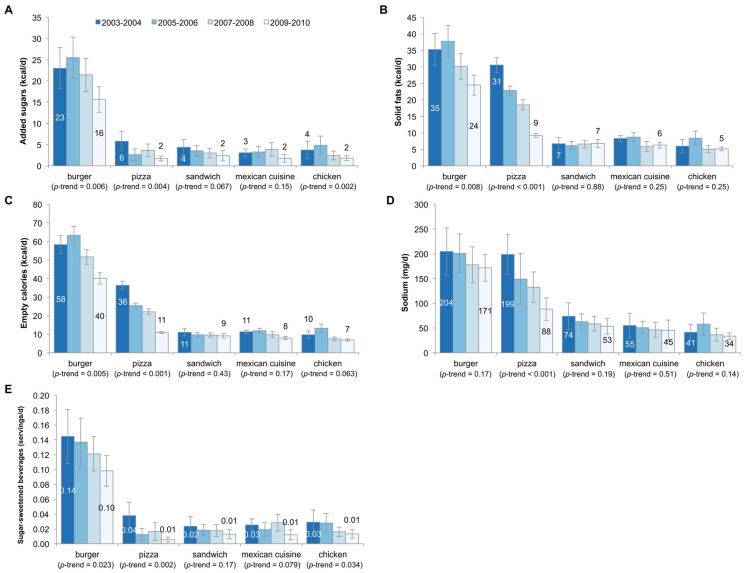
Trends in consumption of added sugars (**A**); solid fats (**B**); empty calories (**C**); sodium (**D**); and sugar-sweetened beverages (**E**) by fast food segment among US children aged 4–19 years, 2003–2010. Error bars are 95% confidence intervals. The serving size for sugar-sweetened beverages is 12 fluid ounces or 355 mL.

Figure 1 shows trends in the consumption of added sugars, solid fats, empty calories, sodium, and SSBs by type of fast food restaurant among the entire population (e.g., per capita consumption). The data are shown for burger, pizza, sandwich, Mexican, and chicken restaurants, as coffee, fish, and Asian fast food were infrequently consumed. Added sugars declined for burger, pizza, and chicken fast food restaurants, while solid fats and empty calories declined significantly from burger and pizza fast food restaurants. Overall, sodium declined significantly for pizza restaurants, but not any other type of fast food restaurant. Sugar-sweetened beverages declined in burger, pizza, and chicken fast food restaurants.

**Figure 2:**
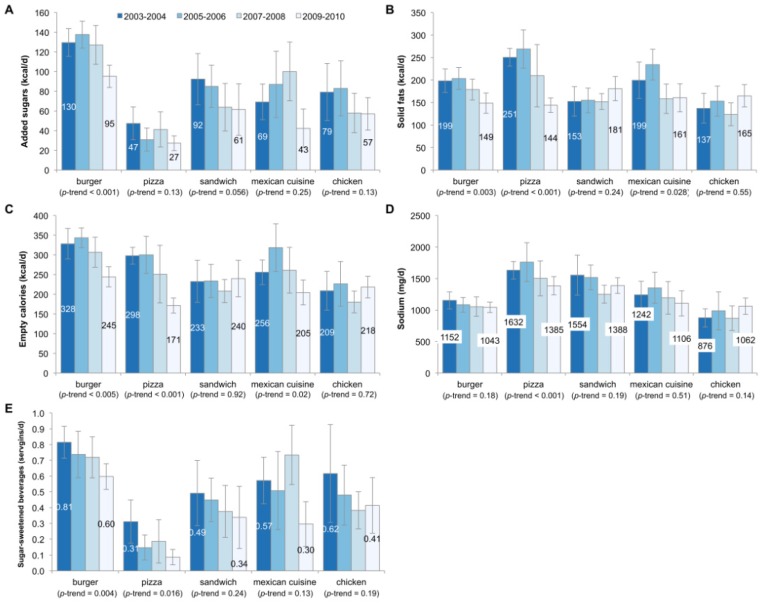
Trends in consumption of added sugars (**A**); solid fats (**B**); empty calories (**C**); sodium (**D**); and sugar-sweetened beverages (**E**) by fast food segment among consumers of each fast food type, US children aged 4–19 years, 2003–2010. Error bars are 95% confidence intervals. The serving size for sugar-sweetened beverages is 12 fluid ounces or 355 mL.

Figure 2 presents similar data, but is limited to consumers of each fast food type. Among burger fast food consumers, added sugars declined significantly from 130 kcal/day to 95 kcal/day; no significant changes were observed for the other market segments. Solid fats and empty calories overall declined significantly for burger, pizza, and Mexican fast food restaurants. Sodium declined significantly for consumers of fast food pizza, but no other market segment. Lastly, SSBs declined significantly from burger and pizza fast food restaurants. 

Trends in fruit consumption were also examined for burger restaurants (data not shown). Fruit was not frequently consumed at other market segments. Total fruit consumption (from whole fruit + fruit juice) from burger restaurants was very low at baseline (0.007 cups/day), but increased to 0.012 cups/day by 2009–2010 (*p*-trend = 0.019). This increase was mostly due to the increase in whole fruit consumption (from nearly zero in 2003–2004 to 0.004 cups/day in 2009–2010, *p*-trend = 0.007). Comparable, but modest increases, in fruit from burger fast food restaurants was observed when restricting the analysis to burger fast food consumers alone.

## 4. Discussion

This descriptive study extends and complements upon prior studies of children’s food consumption away from home, including foods from fast food and full-service restaurants [6]. There are two additional contributions. First, the unique algorithm allowed us to separate FFR foods into distinct market segments, categorized following food industry practice [1,3]. As a result, we were able to evaluate the contribution of burger, pizza, sandwich, Mexican, and chicken restaurants on consumption of added sugars, solid fats, empty calories, sodium, and sugar-sweetened beverages. Second, whereas many previous studies have examined the contribution of specific foods, such as burgers or pizza to children’s diets [2,16], our analyses provided the first insight into nutrients of public health concern sourced from burger or pizza fast food restaurants, themselves [17,18]. The present analyses allow for a more granular examination of the role of the fast food industry in shaping diet quality of US children and youth. 

Our findings, based on nationally representative data, appeared to be consistent with industry reports [19,20]. Between 2003 and 2010, the six largest pizza restaurant chains (Pizza Hut, Domino’s, Papa John’s, Little Caesar’s, Sbarro, and Papa Murphy’s) had a 5.3% decline in total sales. The present data suggest, for children at least, that the decline was due both to fewer visits and reduced sales per visit [1]. By contrast, other FFR segments, including burgers and sandwiches, were not similarly affected [14]; each reporting growth. The present analyses by FFR type opens the door to other opportunities to monitor the impact of industry practices on children’s diet at the population level.

In particular, our approach complements numerous prior studies of menu offerings by FFR chain. Lacking actual consumption data, researchers have turned to analyses of menu offerings to track energy and nutrient content of US chain restaurant menus [21,22,23,24,25,26,27]. However, analyses of menu offerings, when not indexed by sales, are not especially meaningful. The restaurant industry has allowed access to sales data only on rare occasions. One such example was the release of limited sales data by Starbucks to researchers from the Stanford Business School to monitor the impact of calorie labeling in three selected cities [28]. Sales data from other restaurants chains were also made available to researchers to monitor any improvements in food choices following calorie labeling [29,30].

Reducing the amount of solid fats, added sugars, and sodium in the diets of children is a priority for public health nutrition. The present analyses of consumption trends showed that children aged 4–19 years significantly reduced their consumption of solid fats, added sugars, and sodium between 2003 and 2004 and 2009–2010. Grocery stores, FFR and FSRs all contributed to the reduction in added sugars.

The analyses of consumption trends by FFR category have implications for public policy. First, this approach marks a shift from analyses of menu offerings, not indexed by sales, to analyses of FF consumption by children. Second, analyses of fast food consumption trends by FFR type allow for closer monitoring of the impact of menu innovations and other industry practices. As noted above, consumption patterns were very different when expressed per capita versus per consumer. The present analyses provide the first report of consumption trends for solid fat, added sugars, and sodium among children by FFR category.

Perhaps most important, some FFR menu innovations in FFRs appear to have a modest impact on nation-wide food consumption patterns. The introduction of fruit and juices as healthier options is a case in point. While sales data are not available, the present analyses suggest that the consumption of total fruit from burger FFRs rose modestly, but statistically significantly from 2003–2004 to 2009–2010. The increases were largely driven by whole fruit. These results should be interpreted cautiously as the consumption levels were quite low. Interestingly, there are numerous industry reports of more whole fruit being provided/sold by fast food restaurants [31,32], but to our knowledge no published papers. Despite the positive trend there is still considerable room for improvement with regards to the provision of fruit from fast food restaurants. Conversely, the decline in SSB, observed at the national level, was also observed for FFR-sourced beverages among children aged 4–19 years, with the relative decline greater from fast food (relative change of −50%) than from other sources (relative change of −24% from stores and −30% from school). 

While it was not the primary objective of this analyses, these results highlight the primacy of food stores (e.g., grocery stores and other stores, including convenience stores and pharmacies) in providing all dietary constituents at the population level [18]. Consistent with prior findings, stores provided disproportionate amounts of both added sugars and sugar-sweetened beverages per energy compared to other food sources (e.g., fast food and full-service restaurants) [33]. Therefore, while public policy efforts should be explored for all venues, including schools and restaurants, the contribution of store-bought food to dietary intakes cannot be understated and should be addressed by such efforts.

The study had several limitations. First, analyses were based on a 24-h recall, which may result in under-reporting of some foods [34]. A systematic under-reporting of less healthy foods may result in a falsely minimized estimation of energy from desserts, salty snacks, pizza, or soda. Second, the use of proxy responses for young children may have resulted in reported under-consumption of foods when the parent was not present. However, such under-reporting was less likely for FFRs, where parents/guardians were more likely to be present. In addition, the data presented here are somewhat dated, and require updating as new data becomes available. Despite these limitations, this is the first consumption-based study to evaluate the population-wide impact of different FFR segments on the children’s diet.

## 5. Conclusions

Pizza and burger restaurants accounted for much of the decline in solid fats and sodium. The present analyses of consumption time trends by FFR market segment allow us to monitor the evolving quality of FFR foods and to track the impact of menu innovations. In particular, the data showed a drop in FFR SSB, added sugars, and solid fats.

## Figures and Tables

**Table 1:** Estimated means and percent of total (and standard error (SE)) for energy (kcal/day), sodium (mg/day), added sugars (kcal/day), and solid fats (kcal/day) intake by purchase location among children and adolescents, aged 4–19 years, NHANES 2003–2010.

	2003–2004 (*n* = 3400)		2005–2006 (*n* = 3532)		2007–2008 (*n* = 2643)		2009–2010 (*n* = 2803)		
	Mean (SE)	% of Total	Mean (SE)	% of Total	Mean (SE)	% of Total	Mean (SE)	% of Total	*p*-Trend ^a^
**Energy (kcal/day)**									
Total	2195 (20)	-	2119 (35)	-	1999 (23)	-	1990 (25)	-	<0.001
Store	1423 (34)	64.8	1348 (24)	63.6	1274 (24)	63.7	1337 (23)	67.2	0.011
Fast food restaurants	341 (21)	15.6	324 (19)	15.3	276 (15)	13.8	232 (12)	11.7	<0.001
Full-service restaurants	132 (12)	6.0	128 (20)	6.1	113 (14)	5.6	92 (11)	4.6	0.014
School	151 (22)	6.9	131 (11)	6.1	161 (19)	8.1	155 (22)	7.8	0.64
Other	148 (7)	6.7	188 (9)	8.9	176 (14)	8.8	172 (11)	8.7	0.16
**Solid fats (kcal/day)**									
Total	440 (6)	-	444 (9)	-	401 (8)	-	391 (7)	-	<0.001
Store	261 (8)	59.4	259 (6)	58.4	241 (6)	60.0	246 (6)	62.8	0.051
Fast food restaurants	89 (5)	20.2	87 (4)	19.6	68 (5)	16.7	57 (3)	14.4	<0.001
Full-service restaurants	30 (3)	6.8	29 (5)	6.6	24 (3)	6.1	22 (3)	5.4	0.044
School	35 (5)	7.9	31 (3)	6.9	36 (5)	9.0	34 (5)	8.7	0.92
Other	25 (2)	5.8	38 (2)	8.6	32 (4)	8.0	34 (4)	8.7	0.12
**Added sugars (kcal/day) **									
Total	389 (9)	-	360 (12)	-	325 (7)	-	309 (8)	-	<0.001
Store	273 (9)	70.3	245 (9)	68.0	217 (6)	66.8	217 (6)	70.2	<0.001
Fast food restaurants	41 (3)	10.6	42 (4)	11.5	36 (2)	11.0	25 (3)	8.2	<0.001
Full-service restaurants	18 (2)	4.7	17 (3)	4.7	14 (1)	4.2	14 (2)	4.4	0.031
School	18 (3)	4.5	15 (2)	4.2	18 (2)	5.6	17 (2)	5.4	0.98
Other	39 (3)	9.9	41 (3)	11.5	41 (4)	12.5	36 (3)	11.8	0.62
**Sodium (mg/day)**									
Total	3313 (40)	-	3284 (59)	-	3160 (73)	-	3210 (66)	-	0.09
Store	2029 (61)	61.2	1981 (46)	60.3	1913 (49)	60.5	2098 (47)	65.4	0.56
Fast food restaurants	594 (37)	17.9	557 (36)	17.0	482 (29)	15.3	428 (24)	13.3	<0.001
Full-service restaurant	261 (29)	7.9	248 (35)	7.6	231 (38)	7.3	164 (20)	5.1	0.011
School	243 (34)	7.3	216 (19)	6.6	270 (34)	8.5	260 (41)	8.1	0.53
Other	185 (11)	5.6	282 (15)	8.6	264 (26)	8.4	260 (19)	8.1	0.005
**Sugar-sweetened beverages (servings/day) ^b^**									
Total	1.63 (0.07)	-	1.45 (0.09)	-	1.24 (0.06)	-	1.16 (0.06)	-	<0.001
Store	1.12 (0.06)	68.7	1.01 (0.07)	69.8	0.81 (0.04)	65.5	0.85 (0.06)	73.1	<0.001
Fast food restaurants	0.24 (0.02)	14.7	0.19 (0.02)	13.2	0.19 (0.01)	14.9	0.12 (0.01)	10.1	<0.001
Full-service restaurant	0.10 (0.01)	6.0	0.09 (0.02)	6.1	0.07 (0.01)	5.5	0.07 (0.01)	6.1	0.03
School	0.04 (0.01)	2.2	0.02 (0.008)	1.6	0.03 (0.01)	2.2	0.01 (0.004)	0.9	0.037
Other	0.14 (0.01)	8.4	0.13 (0.02)	9.2	0.15 (0.02)	11.7	0.11 (0.01)	9.8	0.27
**Whole fruit (cup equivalents/day) ^c^**									
Total	0.44 (0.04)	-	0.5 (0.02)	-	0.64 (0.06)	-	0.65 (0.04)	-	<0.001
Store	0.35 (0.03)	79.1	0.4 (0.02)	78.9	0.5 (0.06)	78.5	0.49 (0.02)	75.8	<0.001
Fast food restaurants	0.005 (0.002)	1.1	0.01 (0.002)	1.7	0.01 (0.003)	1.6	0.01 (0.001)	1.0	0.36
Full-service restaurant	0.01 (0.002)	1.4	0.01 (0.003)	1.7	0.01 (0.003)	1.1	0.01 (0.002)	1.3	0.62
School	0.04 (0.008)	9.4	0.04 (0.007)	8.2	0.06 (0.008)	9.1	0.08 (0.01)	11.7	0.003
Other	0.04 (0.008)	8.9	0.05 (0.005)	9.5	0.06 (0.014)	9.7	0.07 (0.018)	10.2	0.12
**Fruit juice (cup equivalents /day) ^d^**									
Total	0.57 (0.04)	-	0.48 (0.02)	-	0.44 (0.03)	-	0.45 (0.03)	-	0.008
Store	0.47 (0.04)	83.4	0.39 (0.02)	80.8	0.34 (0.02)	75.9	0.35 (0.03)	78.9	0.003
Fast food restaurants	0.01 (0.002)	1.8	0.01 (0.001)	2.3	0.02 (0.004)	4.0	0.01 (0.002)	3.0	0.077
Full-service restaurant	0.01 (0.002)	1.5	0.01 (0.003)	2.1	0.01 (0.003)	2.1	0.01 (0.002)	1.7	0.70
School	0.04 (0.01)	7.4	0.04 (0.01)	7.5	0.04 (0.01)	9.8	0.04 (0.01)	8.9	0.99
Other	0.03 (0.01)	5.9	0.04 (0.005)	7.3	0.04 (0.01)	8.3	0.03 (0.003)	7.6	0.92

^a^ *p*-trend for absolute amount of energy, sodium or energy from solid fats or added sugars; ^b^ A serving of sugar sweetened beverages is 12 fluid ounces; ^c^ Cup equivalents of commonly consumed fruits include the following amounts: apple (106 g), bananas (136 g), oranges (184 g), and grapes (160 g); ^d^ A cup equivalent of juice is approximately 249 g.

**Table 2:** Estimated means and percent of total (and standard error (SE)) for FFR-sourced energy (kcal/day), solid fats (kcal/day), added sugars (kcal/day), empty calories (kcal/day), sodium (mg/day), sugar-sweetened beverages (servings/day), whole fruit (cups/day), and fruit juice (cups/day) among children and adolescents, aged 4–19 years, NHANES 2003–2010.

	2003–2004 (*n* = 3400)		2005–2006 (*n* = 3532)		2007–2008 (*n* = 2643)		2009–2010 (*n* = 2803)		
	Mean (SE)	% of Total	Mean (SE)	% of Total	Mean (SE)	% of Total	Mean (SE)	% of Total	*p*-Trend ^a^
**Energy (kcal/day)**									
4–11 years	281 (33)	13.7	196 (14)	10.3	201 (22)	10.9	170 (13)	9.4	0.004
12–19 years	400 (22)	17.1	445 (34)	19.1	346 (16)	16.1	293 (19)	13.5	<0.001
**Solid fats (kcal/day)**									
4–11 years	73 (8)	17.4	61 (4)	15.0	53 (5)	13.8	43 (4)	11.6	<0.001
12–19 years	109 (5)	23.9	123 (5)	25.1	95 (5)	22.7	79 (5)	18.7	<0.001
**Added sugars (kcal/day)**									
4–11 years	35 (4)	9.9	30 (4)	9.4	29 (1)	9.4	22 (2)	8.0	0.009
12–19 years	50 (3)	11.8	56 (3)	13.7	52 (5)	14.1	34 (3)	9.2	0.001
**Empty calories (kcal/day)**									
4–11 years	106 (11)	13.6	79 (6)	11.2	79 (9)	11.7	61 (5)	9.6	<0.001
12–19 years	153 (8)	17.4	175 (12)	19.5	127 (7)	16.5	102 (7)	13.3	<0.001
**Sodium (mg/day)**									
4–11 years	421 (44)	15.6	365 (27)	13.5	324 (27)	12.3	309 (24)	11.6	0.018
12–19 years	719 (35)	20.9	775 (38)	22.1	706 (39)	21.0	586 (37)	17.0	0.006
**Sugar-sweetened beverages (servings/day) ^b^**									
4–11 years	0.21 (0.03)	17.4	0.11 (0.01)	13.1	0.14 (0.02)	15.7	0.1 (0.01)	13.7	0.004
12–19 years	0.33 (0.03)	16.0	0.33 (0.04)	16.1	0.27 (0.02)	17.2	0.2 (0.03)	12.6	0.001
**Whole fruit (cup equivalents/day) ^c^**									
4–11 years	0.003 (0.001)	0.6	0.01 (0.003)	1.3	0.02 (0.006)	2.2	0.01 (0.002)	0.7	0.18
12–19 years	0.01 (0.004)	1.7	0.01 (0.003)	2.1	0.01 (0.002)	1.0	0.01 (0.002)	1.4	0.99
**Fruit juice (cup equivalents/day) ^d^**									
4–11 years	0.01 (0.002)	1.1	0.01 (0.002)	1.8	0.02 (0.004)	3.6	0.01 (0.003)	2.2	0.08
12–19 years	0.01 (0.003)	2.5	0.01 (0.002)	2.7	0.02 (0.006)	4.4	0.02 (0.004)	3.9	0.44

^a^ *p*-trend for absolute amount of energy, sodium or energy from solid fats or added sugars; ^b^ A serving of sugar sweetened beverages is 12 fluid ounces or 355 mL; ^c^ Cup equivalents of commonly consumed fruits include the following amounts: apple (106 g), bananas (136 g), orange (184 g), and grapes (160 g); ^d^ A cup equivalent of juice is approximately 249 g.

**Table 3:** Estimated means and percent of total (and standard error (SE)) for FFR-sourced energy (kcal/day), solid fats (kcal/day), added sugars (kcal/day), empty calories (kcal/day), sodium (mg/day), and sugar-sweetened beverages (servings/day) among children and adolescents consuming fast food, aged 4–19 years, NHANES 2003–2010.

	2003–2004 (*n* = 1385)		2005–2006 (*n* = 1475)		2007–2008 (*n* = 988)		2009–2010 (*n* = 979)		
	Mean (SE)	% of Total	Mean (SE)	% of Total	Mean (SE)	% of Total	Mean (SE)	% of Total	*p*-Trend ^a^
Energy (kcal/day)	881 (22)	37.7	858 (21)	36.7	812 (37)	37.3	714 (17)	33.1	<0.001
Solid fats (kcal/day)	229 (6)	47.0	230 (7)	45.1	201 (12)	44.1	174 (6)	39.2	<0.001
Added sugars (kcal/day)	106 (6)	25.0	110 (6)	26.4	106 (6)	27.8	77 (5)	21.3	<0.001
Empty calories (kcal/day)	335 (10)	36.7	340 (9)	37.0	306 (17)	36.7	251 (6)	31.1	<0.001
Sodium (mg/day)	1534 (48)	44.4	1476 (46)	41.8	1422 (81)	42.9	1315 (35)	38.2	<0.001
Sugar-sweetened beverages (servings/day ^a^)	0.62 (0.03)	31.4	0.51 (0.04)	27.0	0.55 (0.04)	34.5	0.36 (0.03)	24.0	<0.001

^a^ A serving of sugar sweetened beverages is 12 fluid ounces or 355 mL.

## Abbreviations

FFR	Fast food restaurant
FSR	Full-service restaurant
MPED	MyPyramid Equivalents Database
NHANES	National Health and Nutrition Examination Survey
